# Application of a wide-range yeast vector (CoMed™) system to recombinant protein production in dimorphic *Arxula adeninivorans*, methylotrophic *Hansenula polymorpha *and other yeasts

**DOI:** 10.1186/1475-2859-5-33

**Published:** 2006-11-14

**Authors:** Gerhard Steinborn, Erik Böer, Anja Scholz, Kristina Tag, Gotthard Kunze, Gerd Gellissen

**Affiliations:** 1 Institut für Pflanzengenetik und Kulturpflanzenforschung, Corrensstr. 3, 06466 Gatersleben, Germany; 2 PharmedArtis GmbH, Forckenbeckstr. 6, D-52074 Aachen, Germany

## Abstract

**Background:**

Yeasts provide attractive expression platforms in combining ease of genetic manipulation and fermentation of a microbial organism with the capability to secrete and to modify proteins according to a general eukaryotic scheme. However, early restriction to a single yeast platform can result in costly and time-consuming failures. It is therefore advisable to assess several selected systems in parallel for the capability to produce a particular protein in desired amounts and quality. A suitable vector must contain a targeting sequence, a promoter element and a selection marker that function in all selected organisms. These criteria are fulfilled by a wide-range integrative yeast expression vector (CoMed™) system based on *A. adeninivorans*- and *H. polymorpha-*derived elements that can be introduced in a modular way.

**Results:**

The vector system and a selection of modular elements for vector design are presented. Individual single vector constructs were used to transform a range of yeast species. Various successful examples are described. A vector with a combination of an rDNA sequence for genomic targeting, the *E. coli-*derived *hph *gene for selection and the *A. adeninivorans*-derived *TEF1 *promoter for expression control of a *GFP *(green fluorescent protein) gene was employed in a first example to transform eight different species including *Hansenula polymorpha, Arxula adeninivorans *and others. In a second example, a vector for the secretion of IL-6 was constructed, now using an *A. adeninivorans*-derived *LEU2 *gene for selection of recombinants in a range of auxotrophic hosts. In this example, differences in precursor processing were observed: only in *A. adeninivorans *processing of a MFα1/IL-6 fusion was performed in a faithful way.

**Conclusion:**

rDNA targeting provides a tool to co-integrate up to 3 different expression plasmids by a single transformation step. Thus, a versatile system is at hand that allows a comparative assessment of newly introduced metabolic pathways in several organisms or a comparative co-expression of bottleneck genes in cases where production or secretion of a certain product is impaired.

## Background

The exploitation of recombinant DNA technology to engineer expression systems for heterologous protein production provided a major task during the last decades. Production procedures had to be developed that employ platforms which meet both the demand for efficient mass production and criteria of safety and authenticity of the produced compounds [1,2]. In this respect, yeasts offer considerable advantages over alternative microbial and eukaryotic cellular systems in providing low-cost screening and production systems for authentically processed and modified compounds. These organisms furthermore meet safety prerequisites in that they do not harbour pyrogens, pathogens or viral inclusions.

The initial yeast system developed for heterologous gene expression was based on the baker's yeast *Saccharomyces cerevisiae*. This platform has been successfully applied to the production of various FDA-approved pharmaceuticals including insulin [3] and HBsAg [4]. However, when using this system, certain limitations and drawbacks are often encountered, since *S. cerevisiae *tends to hyperglycosylate recombinant proteins; N-linked carbohydrate chains are terminated by mannose attached to the chain via an α1,3 bond, which is considered to be allergenic. Other restrictions are the consequence of the limited variety of carbon sources that can be utilised by this species, which limits the fermentation design options. Sometimes, the preferential use of episomal vectors leads to instabilities of recombinant strains; as a result, batch inconsistencies of production runs can be of major concern [2].

Therefore, alternative yeast systems have been defined that can potentially overcome the described limitations of the traditional baker's yeast. Examples include the two methylotrophic yeast species *Hansenula polymorpha *[5,6] and *Pichia pastoris *[5,7], the dimorphic organism *Arxula adeninivorans *[8,9] and others. These organisms share the capability to utilize a broad range of carbon sources, two of them (*H. polymorpha *and *A. adeninivorans*) can assimilate nitrate and are thermotolerant species, with the latter exhibiting a temperature-dependent dimorphism with hyphae formed at elevated temperature and alterations in the extent of O glycosylation. For all systems, a range of host strains and relevant genetic elements are available.

In case of the two methylotrophic species, engineered strains have been developed that exhibit human-like N-glycosylation patterns [6,7,10,11]. The genome of both species has been completely deciphered [6,7,12], and for *H. polymorpha *a microarray chip is available [13]. In case of dimorphic *A. adeninivorans *species, data on chromosomes and on a partial characterization of the genomes are already available [9]. A more detailed description of some of the platforms can be found in [14] and in a recent book on production of recombinant proteins and various chapters therein [1,2,6,7,9,15].

Despite the superior characteristics of yeast hosts in various developments, there is clearly no single system that is optimal for production of all possible proteins. Predictions of a successful development for a given protein can only be made to a certain extent when restricting the initial strain engineering to a single species. The availability of a wide-range yeast vector system enables the assessment of several yeasts in parallel for their capability to produce a particular protein in desired amounts and quality [16-18]. Examples for the application of such vectors are provided in the following overview.

## Results and discussion

### 1. Design and optimization of the CoMed™ vector system

Since vector systems of different yeast species are based on different basic vectors it is very difficult to exchange single cassettes between the yeast systems. To reduce this disadvantage the CoMed™ vector system was established containing the pCoMed™ basic vector for integration of ARS, selection markers, rDNA sequences and expression cassettes. For this purpose, the single modules are flanked by identical restriction sites and are integrated in the same location of the basic vector. In this system, various modules can be integrated. A selection of rDNA elements derived from the *A. adeninivorans *or *H. polymorpha *clusters has been assessed for suitability as targeting sequences. Particular elements of both clusters derived from an ETS-18S – 5.8S segment were found to be optimal. Due to high conservation of the included coding regions targeting of all yeast species is feasible [19]. If for instance the combination of rDNA and the *ALEU2 *gene is chosen, a range of yeasts with this auxotrophy can be targeted. The same holds for the insertion of a dominant selection marker like the *E.coli*-derived *hph *gene conferring resistance to hygromycin B in all yeast species tested so far. The expression cassette is inserted in a final step as fragments derived from pre-constructed plasmids. A range of such cassette elements exists harbouring a promoter of choice, among others the *A. adeninivorans-*derived *TEF1 *promoter mentioned before, and a *S. cerevisiae PHO5 *terminator separated by a multiple cloning site. Again, this promoter was found to be functional in all yeast species tested so far [20,21]. A selection of *ARS *sequences is available that will result in either episomal (*S. cerevisiae*) or chromosomally integrated plasmids (*Hansenula polymorpha*). However inclusion of such a sequence may reduce the range of addressible hosts.

**Figure 1:**
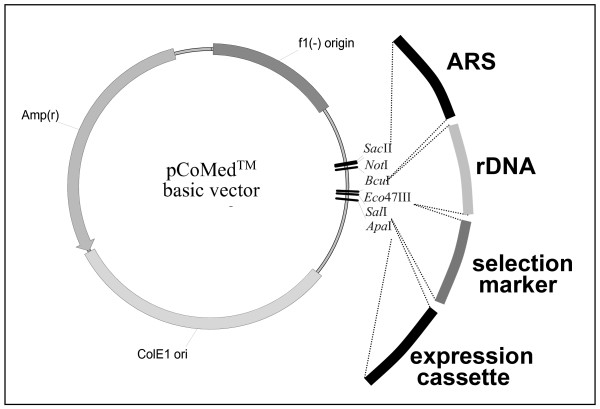
Design and functionality of CoMed™ vector system. The CoMed™ basic vector contains all *E. coli *elements for propagation in the *E. coli *system and a MCS for integration of ARS, rDNA, selection marker and expression cassette modules. For this purpose, *ARS *fragments are flanked by *Sac*II and *Bcu*I restriction sites, rDNA regions by *Bcu*I and *Eco*47III restriction sites, selection markers by *Eco*47III and *Sal*I restriction sites and promoter elements by *Sal*I and *Apa*I restriction sites [38]).

By easy exchange of modules, such a vector can be converted into a plasmid that is optimal for an individual platform, for instance by inserting an expression cassette with a *MOX *promoter element that elicits efficient gene expression in methylotrophic yeasts only. The general design of the plasmid CoMed™ with a selection of components is provided in Fig. 1 and Tab. 1, additional elements and a range of available host strains based on different species can be found in [14]. Variants of this basic vector for the production of antibodies and derivatives thereof are under development. In yet another design, it is possible to linearize the plasmids in a way that leaves behind all bacterial DNA sequences.

**Table 1:** ARS, rDNA regions, selection markers and promoter elements of the CoMed™ vector system.

region/gene	donor organism	reference
ARS		
■ 2 μm DNA	*S. cerevisiae*	[22]
■ ARS1	*S. cerevisiae*	[23]
■ HARS	*H. polymorpha*	[6]
■ SwARS	*Schw. occidentalis*	[24]
		

rDNA region		
■ NTS2-ETS-18SrDNA-ITS1	*H. polymorpha*	[7]
■ 25S rDNA	*A. adeninivorans*	[25]
■ 18S rDNA	*A. adeninivorans*	[19]
■ ITS-5S-ETS-18S-ITS-5,8S-ITS	*A. adeninivorans*	[19]
■ NTS2-ETS-18SrDNA-ITS1	*A. adeninivorans*	[19]
		

selection marker		
■ *URA3*	*S. cerevisiae*	[26]
■ *LEU2*	*S. cerevisiae*	[27]
■ *ALEU2m*	*A. adeninivorans*	[20]
■ *ATRP1m*	*A. adeninivorans*	[29]
■ *HIS4*	*P. pastoris*	[28]
		

expression cassette (promoter)		
■ *FMD *promoter	*H. polymorpha*	[5]
■ *MOX *promoter	*H. polymorpha*	[5]
■ *TPS1 *promoter	*H. polymorpha*	[30]
■ AOX1 promoter	*P. pastoris*	[31]
■ *TEF1 *promoter	*A. adeninivorans*	[32]
■ *AHSB4m *promoter	*A. adeninivorans*	[21]
■ *GAA *promoter	*A. adeninivorans*	[33]
■ *ALIP *promoter	*A. adeninivorans*	[34]
■ *AINV *promoter	*A. adeninivorans*	[35]
■ *AXDH *promoter	*A. adeninivorans*	[36]
■ *RPS7 *promoter	*Y. lipolytica*	[37]

### 2. Wide-range application of the CoMed™ vector system

In a first set of examples, we present the generation of recombinants based on a range of yeast species applying a single vector to transformation. In a first construct, a combination of elements was used that is suited to transform all yeast species tested so far, an rDNA-sequence for wide-range targeting and the *E.coli*-derived *hph *gene as dominant selection marker.

**Figure 2:**
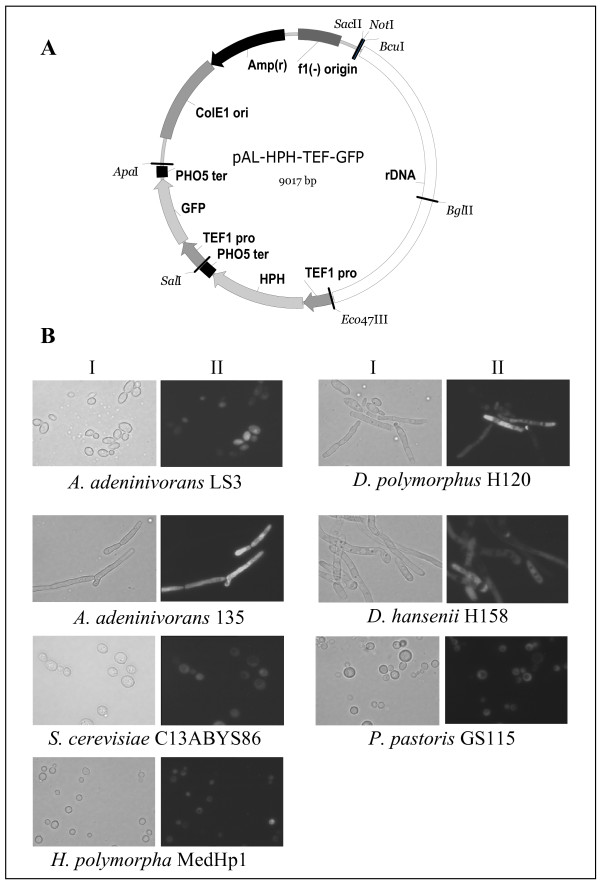
(A) Physical map of the expression/integration vector pAL-HPH-TEF-GFP used in this study. The vector contains the 25S rDNA sequence of *A. adeninivorans *(rDNA, white box) and an expression cassette for the *E. coli*-derived *hph *gene as selection marker in the order *A. adeninivorans-*derived *TEF1 *promoter (TEF1 pro,grey segment), the *hph-*coding sequence (HPH, grey segment), *S. cerevisiae*-derived *PHO5 *terminator (PHO5 ter, black bar). The vector further contains a second expression cassette with *TEF1 *promoter – *GFP *ORF – *PHO5 *terminator elements. The vector contains further an unique *Bgl*II site within the rDNA sequence for linearization. (B) Detection of recombinant GFP-producing yeast cells by fluorescence microscopy. Transformants were cultured for 48 h in YEPD medium at 30°C and subsequently used for fluorescence analysis. (I) transmission, (II) GFP-fluorescence.

For proof of concept, an expression cassette was designed harbouring the *Aequorea victoria-*derived *GFP *gene inserted between the *A. adeninivorans*-derived *TEF1 *promoter and the *PHO5 *terminator of *S. cerevisiae *[18]. An arbitrary selection of yeast species was transformed by the vector linearized within the rDNA targeting sequence, namely *S. cerevisiae*, *A. adeninivorans *(budding cells and filamentous cells), *H. polymorpha*, *Pichia pastoris*, *Debaryomyces polymorphus *and *D. hansenii*. In all transformants, the heterologous DNA was observed to be mitotically stable integrated into the genome of the respective host. For GFP production, the transformants were cultured for 48 h in YEPD medium at 30°C and the produced GFP was detected by fluorescence microscopy. All transformants efficiently produced GFP as shown in Fig. 2. The GFP was distributed within the cytosol and excluded from the vacuoles.

**Figure 3:**
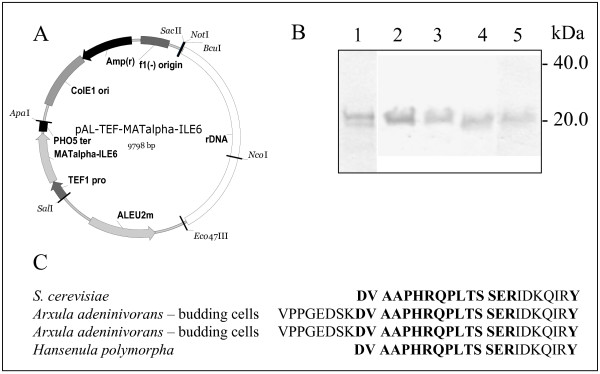
(A) Physical map of the expression/integration vector pAL-ALEU2m-TEF-MATalpha-IL6 used in this study. The vector contains the 25S rDNA sequence of *A. adeninivorans *(rDNA, white box), the selection marker *ALEU2m *(grey segment) and an expression cassette for the *IL6 *gene in the order *A. adeninivorans-*derived *TEF1 *promoter (TEF1 pro, grey segment), the *IL6*-coding sequence, *S. cerevisiae*-derived *PHO5 *terminator(PHO5 ter, black bar) as selection marker. The vector contains a unique *Nco*I site for linearization within the rDNA sequence. (B) IL6 accumulation in recombinant *S. cerevisiae, A. adeninivorans *budding cell and mycelia cultures as well as in *H. polymorpha*. The strains were cultured in YMM supplemented with 2% glucose for 72 h at 30°C (*S. cerevisiae, A. adeninivorans *budding cells, *H. polymorpha*) or 45°C (*A. adeninivorans *mycelia). 20 μl aliquots of culture media were separated on (11%) SDS-PAGE gels, transferred to nitrocellulose filters and probed with anti-IL-6 antibodies. The concentration of recombinant IL-6 was calculated from the signal intensity of a IL-6 standard. (1) IL-6 standard (*E. coli*), (2) *A. adeninivorans *G1211/pAL-ALEU2m-TEF-MATα-IL6 – budding cells (205 mg l^-1^), (3) *A. adeninivorans *G1211/pAL-ALEU2m-TEF-MATα-IL6 – mycelia (144 mg l^-1^), (4) *S. cerevisiae *SEY6210/pAL-ALEU2m-TEF-MATα-IL6 (95 mg l^-1^), (5) *H. polymorpha *HP102/pAL-ALEU2m-TEF-MATα-IL6 (90 mg l^-1^). (C) N-terminus of IL-6 secreted from recombinant *S. cerevisiae, A. adeninivorans *(budding cell and mycelial cultures) and from *H. polymorpha *strains.

In a second construct, we combined an rDNA targeting sequence and an *A. adeninivorans*-derived *LEU2 *[20] for selection. For comparative production, the cytokine IL-6, a secreted protein of industrial relevance, was selected. Recombinant expression systems for IL-6 have been established among others based on *E. coli *[38] and *S. cerevisiae *[39], in addition attempts have been described for *H. polymorpha *[40,41]. In both systems, production is hampered by N-terminal truncation of the product, elicited by a thiol protease that cleaves the mature protein at Arg8 (Arg9 in *E. coli*) [42]. For assessment, we inserted an expression cassette harbouring an ORF for a MFα1/IL-6 fusion protein under control of the elements described before and transformed *leu2 *auxotrophic strains of *A. adeninivorans*, *H. polymorpha *and *S. cerevisiae*. Again, mitotically stable strains were generated. Representatives of the three derived strain collections efficiently secreted the recombinant cytokine into the medium. In this case, product differences could be observed when comparing the secretion products of the different yeast species: the *H. polymorpha *and *S. cerevisiae*-derived molecules were found to be of smaller size than that secreted from the *A. adeninivorans *host. A more detailed comparative MS analysis of tryptic peptides revealed an N-terminal truncation at position Arg8 in *H. polymorpha *and *S. cerevisiae*, but a correctly processed mature IL-6 in *A. adeninivorans *(Fig. 3). This is probably due to the lack of a thiol protease in this dimorphic species. The result emphasizes the need of a careful early pre-selection of a platform for the development of a production process. A detailed description of comparative IL-6 production will be provided elsewhere.

### 3. Wide-range co-integration of different plasmids

It is possible to co-integrate multiple plasmids carrying expressible heterologous genes in the ribosomal DNA by a single transformation step [20,43]. Again, using appropriate selection markers and a functional wide-range promoter for expression control, a comparative single-step integration in a range of selected yeast platforms becomes feasible. In the following section, three promising examples are described.

#### 3.1 Simultaneous introduction of a new metabolic pathway into different yeasts

**Figure 4:**
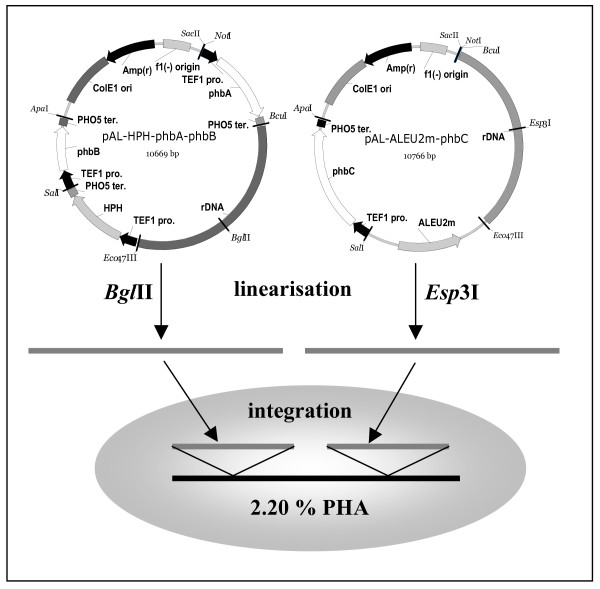
Transformation procedure based on simultaneous integration of the plasmids pAL-HPH-phbA-phbB and pAL-ALEU2m-phbC into the 25S rDNA of *A. adeninivorans *G1211 (*aleu2*). The two plasmids pAL-HPH-phbA-phbB and pAL-ALEU2m-phbC containing the expression cassettes with *phbA, phbB *and *phbC *genes are linearised by *Bgl*II or *Esp*3I digestion, respectively. The resulting fragments flanked by 25S rDNA sequences are co-integrated into the 25S rDNA by homologous recombination. Transformants are selected either by resistance to hygromycin B (plasmid pAL-HPH-phbA-phbB [25,44]) or the complementation of the *aleu2 *mutation (plasmid pAL-ALEU2m-phbC [20]).

In a first example, a new metabolic pathway was introduced into three different yeasts, namely *D. polymorphus*, *D. hansenii *and *A. adeninivorans*. For construction of recombinant biocatalysts, vectors were equipped with the genes *phb*A, *phb*B and *phb*C of the polyhydroxyalkanoate (PHA) biosynthetic pathway of *Ralstonia eutropha *encoding β-ketothiolase, NADPH-linked acetoacetyl-CoA reductase and PHA synthase under control of the *A. adeninivorans*-derived *TEF1 *promoter. Following the previous examples, the vectors were further equipped with an rDNA sequence and the *E. coli*-derived *hph *gene for wide-range integration and selection. Representatives of the three resulting strain collections were found to contain all three heterologous genes as single copies mitotically stable integrated into the genome. In fed-batch cultivations in minimal medium supplemented with 1% ethanol as carbon source, the recombinant yeasts were able to convert efficiently the substrates acetyl-CoA and propionyl-CoA to PHA (2.2% of dry weight) [[9,44]- Fig. 4].

#### 3.2 Construction of an estrogen sensor based on recombinant *A. adeninivorans *cells

**Figure 5:**
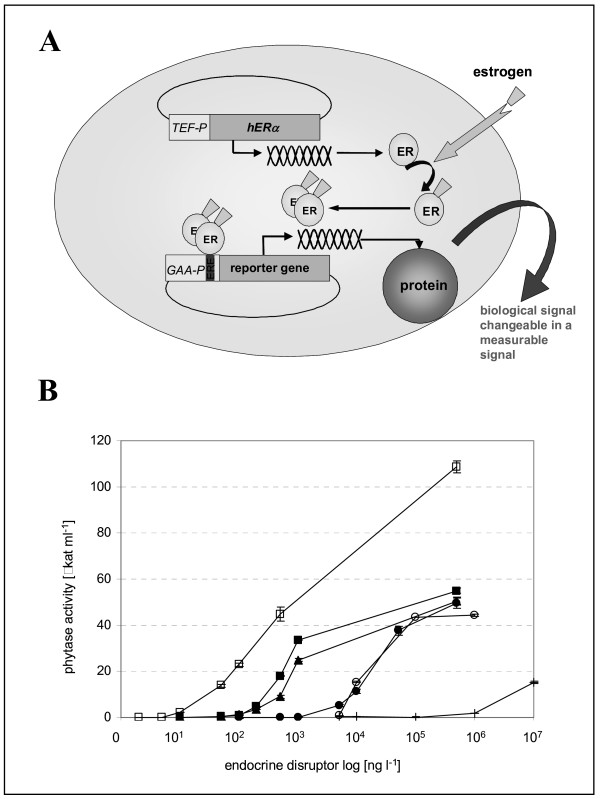
Principle of *A. adeninivorans *yeast estrogen sensor (A-YES). *A. adeninivorans *G1211 transformed with the plasmids pAL-HPH-hERα and pAL-ALEU2m-GAA(2xERE-107)-phyK (G1211/pAL-HPH-hERα – pAL-ALEU2m-GAA(2xERE-107)-phyK) was the bio component of the A-YES. It expresses the estrogen receptor gene (*TEF1 *promoter – *hERα *gene – *PHO5*-terminator) constitutively and produces a relatively constant level of recombinant hERα independent of the estrogen concentration. In the presence of estrogen or estrogen analogues, however, hERα forms a hERα-estrogen-dimer complex, which binds to the ERE-region of the *GAA *promoter located in the second reporter gene expression cassette. The cassette (*GAA-ERE- *promoter – *phyK *gene – *PHO5 *terminator) is activated, the *phyK *gene is expressed and phytase is synthesized. Since this enzyme contains a native signal sequence it is secreted and accumulates extracellularly. The recombinant phytase level is then quantified using a simple biochemical method.(C) Specificity of the A-YES based on *A. adeninivorans *G1211/pAL-HPH-hERα – pAL-ALEU2m-GAA(2xERE-107)-phyK for a range of steroids and steroid metabolites. The graphs depict the log concentration of 17α-ethynylestradiol (□), 17β-estradiol (■), estrone (▲), estriol (●), coumestrol (○) and bisphenol A (+) plotted against the recombinant phytase activity of the medium after 30 h incubation.

Recently, a novel estrogen biosensor based on recombinant *A. adeninivorans *cells has been developed. For this purpose, recombinant *A. adeninivorans *strains were engineered co-expressing the human estrogen receptor α(hERα) and a *Klebsiella*-derived phytase (*phyK*) reporter gene under control of an *A. adeninivorans*-derived glucoamylase (*GAA*) promoter modified by insertion of estrogen-responsive elements (EREs). In response to the presence of estrogenic compounds, hERα dimerizes, and subsequently, reporter gene expression is induced by binding of the newly generated hERα-dimer/estrogen complex to estrogen-responsive elements (ERE) within the promoter. The insertion of different numbers of EREs in three alternative promoter positions and its effect on reporter gene expression were assessed. In a particular construct, a detection limit of 5 ng l^-1 ^and a quantification limit of 10 ng l^-1 ^for 17β-estradiol-like activity could be achieved. A convenient photometric assay enables estrogen monitoring in sewage samples within 30 hrs ([45] – Fig. 5). As the two final plasmids are designed for wide-range transformation a range of other yeast species can now be assessed for this application.

#### 3.3 Assessment of secretory pathway genes for the production of IFNγ

IFNγ is produced by CD4 and CD8-positive T and NK (natural killer) cells. The mature protein consists of 146 amino acids. IFNγ is a potent anti-viral and anti-parasitic agent. It has been assessed for treatment of opportunistic infections in AIDS patients, for treatment of eosinophilia in severe atopic dermatitis and for treatment of osteopetrosis [46,47]. Two active forms of 25 and 20 kDa exist, differing in the extent of glycosylation. Glycosylation is not required for biological activity [48] but for proteolytic stability [49]. It is therefore desirable to have access to an efficient expression platform in which a cytokine can be produced as glycosylated protein. The protein has been produced in *E. coli *[50,51] and in mammalian cells [52].

**Figure 6:**
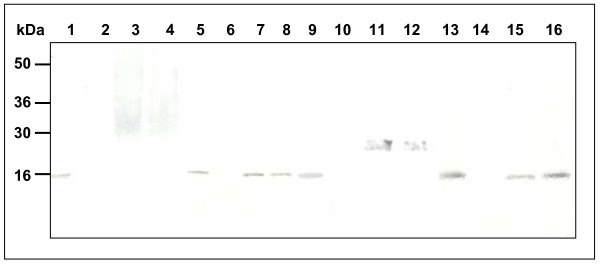
Glycosylation analysis of secreted IFNγ. A strain of the pMrL-IFNg/pMrL-CNE collection was cultured on a 500 ml scale. The proteins of the supernatant were precipitated with (NH_4_)_2_SO_4_. An aliquot of the precipitate was digested with PNGase F as described in Materials and methods. Untreated and PNGase F-treated protein samples were compared to isolates from a previous strain collection. The various samples were separated by SDS-PAGE; proteins were visualized by immunoblotting as described before. (1,5,9,13) IFNγ standard (*E*.*coli*), (2,6,10,14) *H. polymorpha*, (3,4) *H. polymorpha*/CoMed8-MATα-IFNγ, (7,8) *H. polymorpha*/CoMed8-MATα-IFNγ – deglycosylated, (11,12) *H. polymorpha*/CoMed8-MATα-IFNγ – CoMed14-CNE, (15,16) *H. polymorpha*/CoMed8-MATα-IFNγ – CoMed14-CNE – deglycosylated.

IFNγ is poorly secreted from recombinant *H. polymorpha *cells in the form of hyperglycosylated molecules. In other examples, overexpression of secretory pathway genes has been shown to improve both, the yield and the quality of a recombinant product in several yeast platforms. Examples include the overexpression of *KAR2 *[53], *PDI *[54], *SSO1/2 *[55], *CMK2 *[56] and *KEX2 *[57]. However, it became apparent that the observed improvements were restricted to a specific recombinant product development in meeting the demands to overcome a particular limitation in the respective recombinant strains. When encountering such limitations like poor secretion, impaired processing or glycosylation, it seems advisable to assess several candidate genes of the secretion machinery or even combination thereof for an enhanced probability of success. A selection of four secretory pathway genes was assessed for possible impact on secretion improvements in this case. For assessment, wide-range rDNA integration vectors harbouring the *H. polymorpha*-derived *KAR2, PDI, SSO2 *or *CNE1 *genes [58] were co-integrated along with the IFNγ integration/expression vector. Overexpression of *CNE1 *resulted in an increased secretion of the cytokine predominantly consisting of molecules of distinct size. Deglycosylation with PNGaseF resulted in a M_r _reduction of the secreted IFNγ corresponding to the removal of two N-linked glycoside chains. Coexpression of *KAR2, PDI1 *and *SSO2 *exhibited no effect (Fig. 6). Again, co-integration into alternative yeasts is now feasible. Furthermore a co-integration of the *IFN*γ gene with more than a single secretory pathway gene can be envisaged.

## Conclusion

The CoMed™ vector system is a versatile system built up in a modular way. Modules comprise of ARS sequences, rDNA targeting sequences and dominant or complementation selection markers. For expression cassettes, a choice of promoters from various sources is available, separated in the module by a MCS from an *S. cerevisiae*-derived *PHO5 *terminator.

A combination of an rDNA integration sequence and a suitable selection marker enables transformation of host strains derived from a wide range of yeast species. The vectors can easily be transformed into traditional species-specific vectors.

A single type of plasmid or multiple types of plasmids can simultaneously be integrated into the genome of the various hosts.

The vector system thus provides a powerful tool to transform several yeasts in parallel at an early stage of a particular process development thereby avoiding potential cost- and time-consuming failures.

## Materials and methods

### Strains and media

*E. coli *TOP 10 [F', *mcrA*, Δ(*mrr-hsdRMS-mcrBC*), Φ80 Δ*lacZ*-Δ*DM15*, *nupG*, Δ*lacX74*, *deoR*, *recA1*, *araD139*, Δ(*ara,leu*), *7697*, *galU*, *galK*, λ^-^, *rpsL*, *endA1*] from Invitrogen, USA, served as host strain for bacterial transformation and plasmid isolation. Strain was grown in LB medium supplemented with ampicillin (50 μg ml^-1^; AppliChem, Germany) when required for selection.

The yeast strains *A. adeninivorans *LS3 [59], *A. adeninivorans *135 [32], *A. adeninivorans *G1211 ([*aleu2 *– [60]), *D. hansenii *H158 (provided by the strain collection of UFZ, Leipzig/Germany), *D. polymorphus *H120 (provided by the strain collection of UFZ, Leipzig/Germany), *P. pastoris *GS115 (*his4 *– Invitrogen/USA) and the *H. polymorpha *MedHp1 (*odc1 *– [14]) as well as *S. cerevisiae *C13ABYS86 (MATα *leu2 ura3 his pra1 prb1 prc1 cps *– [61]) were used as hosts. All strains were grown either under non-selective conditions in complex medium (YEPD) or under selective conditions in a yeast minimal medium (YMM) supplemented with 2% of a selected carbon source [62,63]. Cultivation was performed at 30°C.

Agar plates were prepared by adding 1.6% (w/v) agar to the media. Hygromycin B (Roche Diagnostics, Germany) was added as 150 – 400 μg ml^-1 ^when required for selection.

### Yeast transformation

*A*.*adeninivorans *LS3, *A*.*adeninivorans *135, *A. adeninivorans *G1211, *D*.*hansenii *H158, *D.polymorphus *H120, *H. polymorpha *MedHp1, *P. pastoris *GS115 and *S. cerevisiae *C13ABYS86 were transformed according to [25,64]. Stable transformants were obtained after a sequence of passages on selective and non-selective media. After transformation of plasmids with the *hph *selection marker, hygromycin B-resistant colonies were selected on YEPD agar plates supplemented with 150 – 400 mg l^-1 ^hygromycin B (200 mg l^-1 ^for *A.adeninivorans *LS3 and 135, 250 mg l^-1 ^for *D*.*hansenii *H158 and *D*.*polymorphus *H120, 400 mg l^-1 ^for *H. polymorpha *MedHp1, 150 mg l^-1 ^for *P. pastoris *GS115and *S. cerevisiae *C13ABYS86). Single colonies were isolated and grown on YEPD medium and hygromycin B at 30°C for 2 days. This step was repeated three times before the cells were plated on non-selective YEPD agar and grown for 3–5 days at 30°C. A single colony from each transformant was isolated and defined as a strain.

In case of auxothrophy complementation the transformants were selected on YMM agar plates lacking the respective amino acid.

### Isolation and characterisation of nucleic acids

Plasmid DNA, restriction fragment isolation, labeling of fragments and Southern transfer were carried out as previously described by [32,65].

### Protein analysis

SDS-PAGE with 6 μg cell extract protein and Western blots were performed as described by [66]. The dye binding method of Bradford [67] was used for quantitative determination of protein concentration in cell extract with bovine serum albumin as a standard protein. IL-6 and IFNγ were immunologically detected by Western blot analysis using specific anti-IL-6 or IFNγ antibodies (R&D systems/USA) for detection. Blots were stained with Western Blue Stabilized Substrate (Promega, Germany).

IFNγ samples were digested with PNGaseF (Boehringer, Mannheim) following the instructions of the supplier. Untreated and digested samples were separated and visualized as described before.

### Fluorescence microscopy

GFP expression was visualized in yeast cells by fluorescence microscopy (Axioskop, Zeiss Jena, Germany; excitation at 470 nm and detection using the BP500–530 nm emission filter). These conditions allowed visualization of GFP-mediated fluorescence avoiding a significant auto-fluorescence background. Single images (512 × 512 pixels) were collected using line-averaging eight times, each for 1 s.

### Measurement of yeast dry mass and PHA

PHA, detected using gas chromatography/mass spectrometry, and yeast dry mass were measured as described by [68,69].

### Estrogenic activity assay

*A. adeninivorans *G1211 transformed with the plasmids pAL-HPH-hERα and pAL-ALEU2m-GAA(xERE)-phyK (G1211/pAL-HPH-hERα – pAL-ALEU2m-GAA(xERE)-phyK) was the bio component of the assay. These cells were cultured in YMM with 2% glucose at 30°C for 48 h and stored as 50 μl aliquots. For detection of estrogenic activity, 950 μl of the samples were supplemented with 2% maltose (final concentration) and 50 μl of stationary yeast cell suspension (final OD_600 nm _= 1) was added. The suspension was cultured for 30 h at 30°C on a shaker.

Subsequently all samples were centrifuged for 10 min at 5000 × g to separate the cells from the cultivation medium. The extracellularly accumulated phytase activity was assessed through a modified method initially described by [70,71]. Aliquots (25 μl) of the supernatant samples were incubated with 25 μl 0.1 M sodium-citrate (pH 3.9) containing 3.5 mM 4-nitrophenyl phosphate for 60 min at 37°C in microtitre plates. The reaction was stopped by adding 50 μl 15% TCA. After adding of 100 μl 1 N sodium hydroxide, 4-nitrophenol was measured at 405 nm with the reader "Sunrise" from Tecan (USA) [45].

## Abbreviations

CoMed is a trade name (abbreviation of Construction set PharmedArtis).

## Competing interests

The author(s) declare that they have no competing interests.

## Authors' contributions

GS, EB, AS and KT carried out the experiments and participated in draft a document describing the work at hand. GK and GG designed the work, completed the experiments, and wrote the manuscript. All authors read and approved the final manuscript.
